# miR-147b-modulated expression of *vestigial* regulates wing development in the bird cherry-oat aphid *Rhopalosiphum padi*

**DOI:** 10.1186/s12864-020-6466-7

**Published:** 2020-01-22

**Authors:** Yinjun Fan, Xiuxia Li, Abd Allah A. H. Mohammed, Ying Liu, Xiwu Gao

**Affiliations:** Department of Entomology, China Agricultural University, No. 2 Yuanmingyuan West Road, Haidian District, Beijing, 100193 People’s Republic of China

**Keywords:** Wing development, *Vestigial*, MicroRNA, *Rhopalosiphum padi*

## Abstract

**Background:**

Most aphids exhibit wing polyphenism in which wingless and winged morphs produce depending on the population density and host plant quality. Although the influence of environmental factors on wing polyphenism of aphids have been extensively investigated, molecular mechanisms underlining morph differentiation (i.e. wing development /degeneration), one downstream aspect of the wing polyphenism, has been poorly understood.

**Results:**

We examined the expression levels of the twenty genes involved in wing development network, and only *vestigial (vg*) showed significantly different expression levels in both whole-body and wall-body of third instar nymphs, with 5.4- and 16.14- fold higher expression in winged lines compared to wingless lines, respectively in *Rhopalosiphum padi*. *vg* expression was higher in winged lines compared to wingless lines in third, fourth instar nymphs and adults. Larger difference expression was observed in third (21.38-fold) and fourth (20.91-fold) instar nymphs relative to adults (3.12-fold). Suppression of *vg* using RNAi repressed the wing development of third winged morphs. Furthermore, dual luciferase reporter assay revealed that the miR-147 can target the *vg* mRNA. Modulation of miR-147b levels by microinjection of its agomir (mimic) decreased *vg* expression levels and repressed wing development.

**Conclusions:**

Our findings suggest that *vg* is essential for wing development in *R. padi* and that miR-147b modulates its expression.

## Background

Phenotypic plasticity is prevalent in organisms [1]. Polyphenism is an extreme case of phenotypic plasticity in which discrete phenotypes are produced from the same genotype [2]. Most aphids exhibit wing polyphenism in which winged and wingless morphs are produced depending on environmental stimulus (e.g. population density and host nutrition) during parthenogenetic generations [3]. The wingless morphs maximize reproduction, allowing rapid colony growth. In contrast, the winged morphs engage in dispersal which enable them to seek out new habitats, mates, and food resources [4]. Generally, wing morphs include determination and differentiation processes that occur at completely different times during aphid development. Mostly, morph determination occurs during embryogenesis in the maternal ovary in response to environmental cues perceived by the mother. Morph differentiation (i.e. wing development/degeneration) occurs during postembryonic development [5]. The influence of external cues on wing dimorphism of aphids have been extensively investigated, and some studies indicated that neuroendocrine signaling pathways regulate wing morph determination [6, 7]. For example, recently, ecdysone signaling was found to be critical for controlling wing morph determination in *Acyrthosiphon pisum* [8].

The bird cherry-oat aphid, *Rhopalosiphum padi* (L.), is one of the most globally abundant cereal aphid pests. In addition to directly feeding on plants, *R. padi* damages cereal crops by transmitting *barley yellow dwarf virus*, which causes cereal losses of between 20 to 80% [9–11]. *R. padi*, like most aphids, can produce wing morphs when experiencing the crowding and poor nutrition conditions [12, 13].Winged morphs play an important role in long distance migration and host alternation processes. Winged individuals may carry viruses in autumn, which are considered as a major epidemiological factor for determining the disease incidence [14–16]. *R. padi* has holocyclic and anholocyclic life cycles, and it can overwinter anholocyclically where winters are mild or the absence of the primary host (Prunus L.) [17, 18]. Climate change and urbanization has been suggested an increase of anholocyclic clones and winged individuals, causing more serious virus transmission and cereal damage [19–21]. To date, the control of *R. padi* relies on the application of chemical insecticides, which have leaded to insecticide resistance and environmental pollution [22]. Therefore, understanding the molecular mechanisms of wing development process is important for controlling *R. padi* effectively.

It is well established that wing development in the parthenogenetic aphids is the default development pathway. Specifically, all aphids are born through viviparous reproduction with wing primordia, and it degenerates by the second instar in the unwinged morph [23]. In the winged, the wing primordia continue to slowly grow through the first three nymphs, and they are well developed in the fourth instar [24, 25]. Gene networks underlying the wing patterning, growth and differentiation (we will refer to these as “wing development” for simplicity in the study) have been well investigated in *Drosophila melanogaster.* Principal wing development gene homologs are largely conserved across insects [24, 26]. In *A. pisum*, the expression levels of 11 genes involved in wing development were investigated between wing morphs, and only one gene (i.e. *apterous)* was found to exhibit significantly high expression level [24]. Therefore, the goal of the current study is to improve the understanding of whether wing development genes contribute to wing development or degeneration in *R. padi*. Here, we depict a gene network involved in major wing development events deduced from *D. melanogaster* including anterior-posterior (A-P) patterning genes such as *engrailed* (*en*), *hedgehog* (*hh*), *decapentaplegic* (*dpp*), *brinker* (*brk*), *optomoter-blind* (*omb*), *spalt-major* [27] [28], dorsal-ventral (D-V) patterning genes such as *apterous* (*ap1, ap2*), *Notch* (*N*), *serrate* (*ser*), *delta* (*dl*), *suppressor of hairless* (*su* (*h*)), *wingless* (*wg*), *distalless* (*dll)*, *scalloped* (*sd*), and *vestigial* (*vg*) [29], a wing hinge development gene *homothorax* (*hth*) [30, 31], a Hox gene *ultrabithorax* (*Ubx*) [32], a wing notch and blade differentiation gene *extradenticle* (*exd*) [31], and a wing intervein development gene *serum response factor* (*srf*) [33]. Next, we investigated the expression levels of the 20 genes in whole bodies and body walls (enriching in tissues containing cells forming the wings in winged lines) of wing morphs at third instar nymphs (the earliest stage for distinguishing wing morphs in outer morphology) in *R. padi*, and only *vg* showed significantly different expressions in both cases. The role of *vg* in wing development in *R. padi* was further investigated by *vg* RNAi. Also, our results reveal that the expression of *vg* is regulated by miR-147b. These findings provide evidence that *vg* mediated by miR-147b regulates wing development in *R. padi*.

## Results

### Expression profiles of wing development genes in wing morphs

**Fig. 1:**
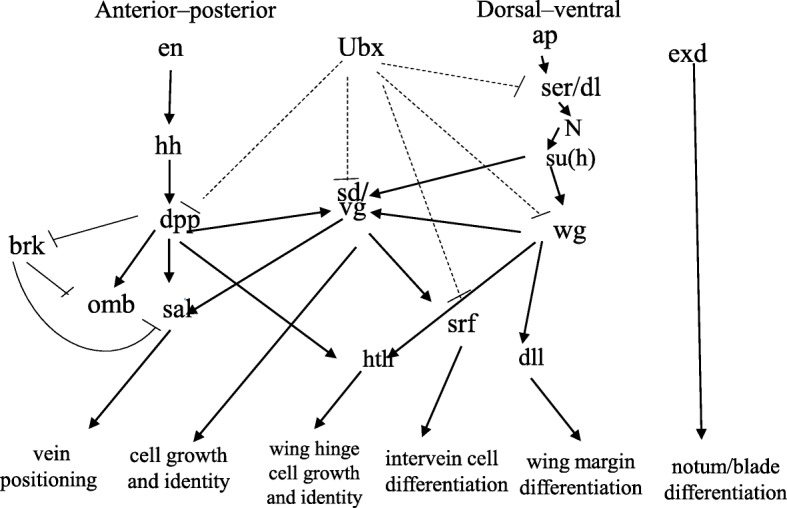
The presumptive wing-patterning network in *Drosophila* [24, 26]. The aphid orthologs of these *Drosophila* genes were examined in this study. Abbreviations*: en*, *engrailed*; *hh*, *hedgehog*; *dpp*, *decapentaplegic*; *brk*, *brinke*r; *omb*, *optomoter-blind*; *sal*, *spalt-major*; *Ubx*, *ultrabithorax*; *sd*, *scalloped*; *vg*, *vestigial*; *ap*, *apterous*; *ser*, *serrate*; *dl*, *delta*; *N, notch*; *su* (*h*), *suppressor of hairless*; *wg*, *wingless*; *dll*, *distalless*; *hth*, *homothorax*; *srf*, *serum response facto*r; *exd*, *extradenticle*. Dashed lines indicate regulatory interactions specific to the hindwing disc. Arrowheads and bars indicate activation and repression, respectively

**Fig. 2:**
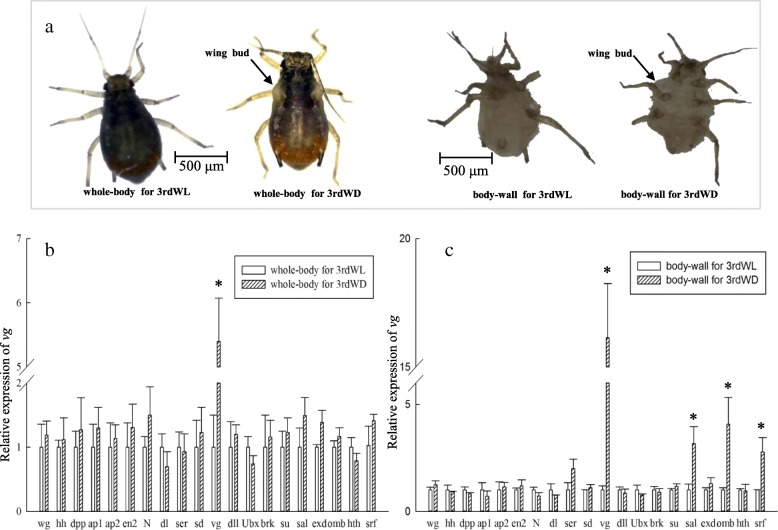
Expression profiles of wing patterning genes between wingless (WL) and winged (WD) third instar nymphs of *R. padi*. **a** Phenotypes of whole body and body walls of third nymphal instar wing morphs in *R. padi*. Expression profiles of wing patterning genes in whole bodies (**b**) and body walls (**c**). Data are means ± SD. Asterisks indicate significance according to Student’s t-test (*P* < 0.05)

To determine which genes may be involved in wing differentiation during post-embryonic development in *R. padi*, we evaluated the expression levels of twenty known wing development genes (Fig. 1) between wingless and winged third instar nymphs using qRT-PCR. All genes had similar expression levels between wingless and winged whole bodies except for *vg*, in which expression was 5.4-fold higher in the whole bodies of winged aphids than in the wingless aphids (Fig. 2b). Expression levels of *vg*, *sal*, *omb* and *srf* were 16.14-, 3.16-, 4.07- and 2.77-fold higher in body walls of winged aphids relative to wingless aphids, respectively (Fig. 2c).

### Expression patterns of *vg* in wing morphs

**Fig. 3:**
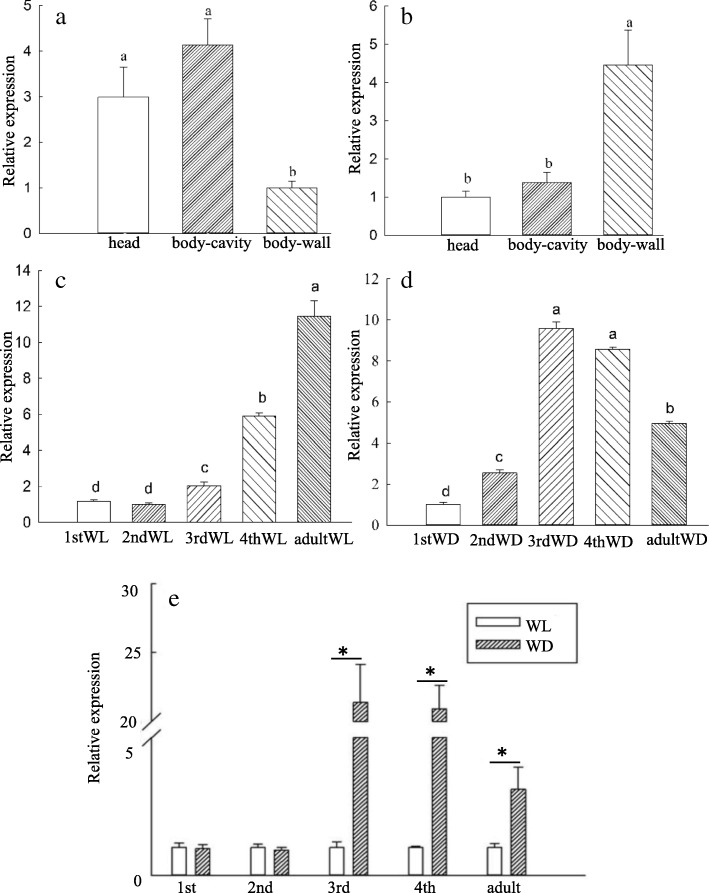
Expression profiles of *vg* in different body parts, developmental stages, and wing morphs of *R. padi*. *Vg* expression levels in different tissues of third instar wingless (**a**) and winged morphs (**b**). Expression levels of *vg* in wingless (**c**) and winged lines (**d**) during development. **e** Comparison of *vg* expression between wing morphs during developmental stages. Abbreviations: first (1st), second (2nd), third nymphal instars (3rd), and fourth (4th) instar nymphs, wingless (WL), winged (WD). Different letters on the histogram bars indicate significant differences based on one-way analysis of ANOVA followed by Tukey’s HSD multiple comparison test (*P* < 0.05). Asterisks indicate significant difference according to Student’s t-test (*P* < 0.05)

The expression patterns of *vg* were further determined in different tissues of the third instar nymphs and different developmental stages. The results showed that the expression levels of *vg* were the lowest in the body wall of the third instar wingless aphids (Fig. 3a), and the highest in the body wall of the third instar winged aphids (Fig. 3b). The expression levels of *vg* were stable from the first to the second nymph stage, then increased sharply from the third nymph to the adult stage in the wingless morphs (Fig. 3c). In contrast, *vg* expression increased from the first to the third instar nymphs and then decreased in the adult stage in the winged morphs (Fig. 3d). Altogether, the highest expression of *vg* was found in the third instar nymphs, and it was 9.58-fold higher relative to the first instar nymphs, during winged nymph development.

Comparing the expression levels of *vg* between wingless and winged body walls with the development stages revealed interesting trends. The expression levels of *vg* were higher in winged aphids than in wingless aphids in the third and fourth instar nymphs as well as in adults, and higher difference ratios were observed in third (21.38-fold) and fourth (20.91-fold) instar nymphs compared with the adult (3.12-fold) between wing morphs. However, the expression levels of *vg* had no significant difference in the first and second instar nymphs between wing morphs (Fig. 3e).

### Conserved domains of *vg* and expression of VG protein

We obtained the full length 2471-bp *vg* cDNA that included a 456-bp 5′-untranslated region (5’UTR), a 956-bp 3’UTR and an open reading frame (ORF) of 1059-bp. The ORF encodes 670 amino acids with a predicted molecular weight of approximately 39 kDa. The cDNA sequence has been deposited in GenBank under the accession number MH168385. The VG protein contains the Vg_Tdu domain, which is highly conserved among holometabolous and hemimetabolous insects (Additional file 1: Figure S1).

To determine whether the VG protein had different expression between wing morphs as *vg* mRNA did, we investigated the VG protein expression levels between wing morphs third instar body walls. The result showed that there were higher levels of the protein in the body wall of winged aphids relative to wingless aphids (Additional file 1: Figure S2).

### RNAi knockdown of *vg* suppresses wing development

**Fig. 4:**
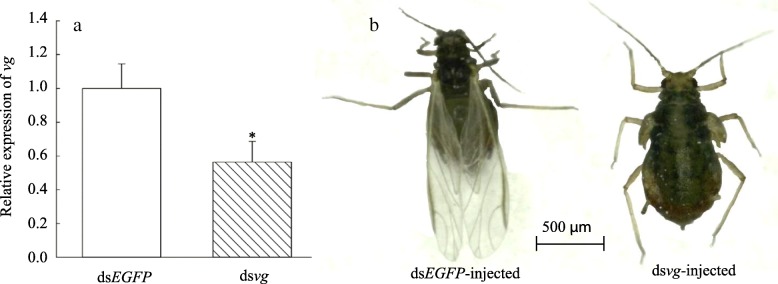
RNAi of *vg* and its effects on wing development of *R. padi.* (A) RNAi-mediated suppression of vg transcripts in third instar winged aphid injected with dsRNA-*vg* for 24 h. (B) Phenotypes of third nymphal instars winged aphid after injected with dsRNA of *vg* for 48 h. Asterisks indicate significant difference according to Student’s t-test (*P* < 0.05)

RNAi experiments were performed to understand the relationship between wing development and *vg* gene expression. Third instar aphids of the winged lines were injected with dsRNA. The mortality was 30% (dsRNA) and 27% (dsEGFP) at 24 h following injection (*n* > 100). In addition, at 24 h after injection with *vg* dsRNA, the mRNA levels of *vg* decreased significantly by 44% compared to control insects injected with dsEGFP (Fig. 4a). After 48 h, 68% aphids injected by *vg* dsRNA (n~ 20) had under-developed wings compared to the dsEGFP control aphids, which were 100% normal (Fig. 4b).

### miR-147b putatively regulates the expression of *vg*

To determine whether the differently expressed *vg* between wing morphs resulted from the *vg* DNA copy numbers, we investigated the *vg* DNA expression levels between body walls of third instar aphids. There was no significant difference in *vg* DNA expression levels between wing morphs (Additional file 1: Figure S3).

**Fig. 5:**
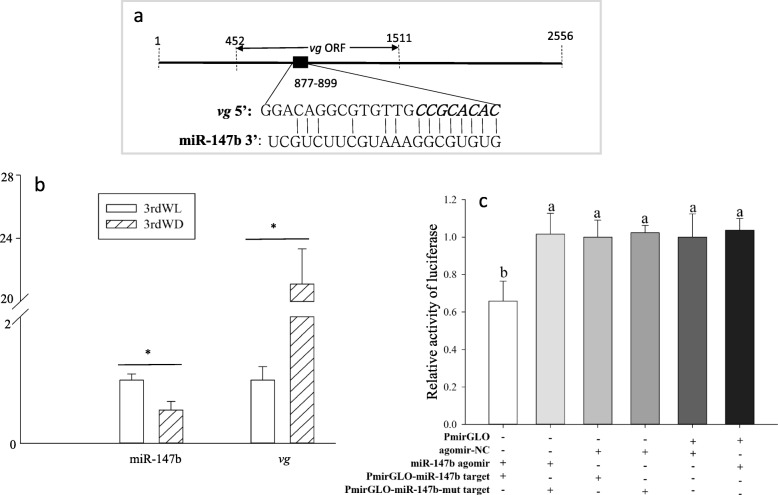
Functional validation of the miR-147b target sites in *vg*. **a** Predicted sites of miR-147b in the ORF of *vg.* The nucleotides in bold italics were mutated for TATACAGT in the PmirGLO-miR-147b-mut target. **b** Relative expression levels of miR-147b and *vg* in the body walls of third wingless (3rdWL) and winged (3rdWD) lines*.* **c** Luciferase reporter assays were performed by co-transfecting the miR-147b agomir with a luciferase reporter gene linked to the *vg* targets. Different letters on the histogram bars indicate significant differences based on one-way ANOVA followed by Tukey’s multiple comparison (*P* < 0.05). Asterisks indicate significant difference based on Student’s t-test (*P* < 0.05)

miRNA prediction showed a predicated target site of miR-147b that was found in bases 877 to 899 of the ORF of *vg* with a high complementarity (Fig. 5a). The transcriptional levels of miR-147b in winged aphids were significantly lower than in wingless aphids, and the opposite effect was observed in the expression levels of *vg* (Fig. 5b). Because aphid wing polyphenism is associated with colony density, we examined the effect of density on the expression levels of *vg* and miR-147b in third instar nymphs of the wingless morph. No significant differences in *vg* and miR-147b expressions were observed between the body walls of third instar wingless lines from LD and HD conditions (Additional file 1: Figure S4).

To determine whether miR-147b can bind to *vg* mRNA, the predicted target sequences of *vg* were inserted into the pmirGLO vector to construct the recombinant vector pmirGLO-miR-147b. Firefly luciferase activity normalized against *Renilla* luciferase was significantly reduced when pmirGLO-miR-147b was co-transfected with the miR-147b agomir (mimic). However, the luciferase activity levels of the pmirGLOmiR-147b-mut construct were not dramatically affected by the miR-147b agomir compared with the unmutated constructs (Fig. 5c). These results suggest that miR-147b binds to the target sequence in the *vg* mRNA.

### miR-147b can modulate wing development

**Fig. 6:**
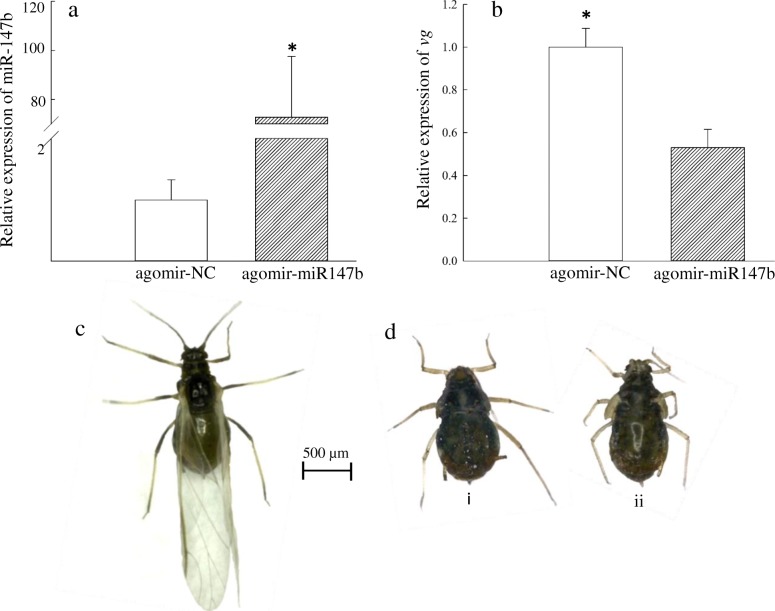
The effect of miR-147b on wing development. The expression levels of miR-147b (**a**) and *vg* (**b**) in third instar nymph winged lines after injection of miR-147b agomir for 24 h, resepectively. Phenotypes of third nymphal winged aphid after injecting with agomir-NC (**c**) and miR-147b agomir (**d**) for 48 h. (i) and (ii) phenotypes are at rates of 75 and 25%, respectively. Asterisks indicate significant difference based on Student’s t-test (*P* < 0.05)

To verify whether the expression of *vg* is regulated by miR-147b, miR-147b agomir was injected into the winged third nymphs of *R. padi*, and we examined the expressions of miR-147b and *vg* after 24 h, respectively. The mortality was 28% (miR-147b agomir) and 22% (agomir-NC) at 24 h following injection. Compared with control group, expression levels of *vg* were decreased by 47% after injection for 24 h (Fig. 6b). Wing development was dramatically repressed in the group injected with the miR-147b agomir, which exhibited two types of phenotypes at rates of 75 and 25% (n~20), respectively (Fig. 6d); however, wing development in the control group injected with the miRNA negative control was normal at rates of 100% after 48 h (Fig. 6c). These results demonstrated that miR-147b can affect *vg* expression and modulate wing development.

## Discussion

It is well known that the *vg* gene plays a central role in the development of the *Drosophila* wing [35]. In the absence of *vg* gene expression, cells within the larval wing and haltere imaginal discs fail to proliferate normally [36]. Although there are few studies describing the role of *vg* in wing development in other insects, principal wing development gene homologs are very conserved among insects [24, 37]. Our results showed that only *vg*, out of 20 genes involved in major wing development events (Fig. 1), had higher expression levels in both the whole body and the body wall of winged lines, compared to wingless morphs, in *R. padi* (Fig. 2). The expression of *vg* had a greater difference in body-wall (16.14-fold) than that in whole-body (5.4-fold) between the winged and wingless morphs (Fig. 2). *Vg* expression levels were the highest in the body wall of the winged lines, while they were the lowest in the body wall of the wingless lines. This pattern of expression suggests *vg* plays a key role in wing development in aphids. The point was subsequently verified by *vg* dsRNA which depressed wing development of third winged aphids (Fig. 4). Higher expression of *sal* and *srf* were also found in the body wall of winged aphids compared to wingless aphids (Fig. 2c). This difference indicate that *vg* may regulate their expression, because the *sal* and *srf* is the downstream of *vg* in *Drosophila* (Fig. 1) [37]. Also, *Omb* was expressed at higher levels in winged aphids relative to wingless aphids (Fig. 2). Whether increased expression of *omb* results from higher levels of *vg* in winged aphids is unknown. The other 16 genes had no significant morph effect in third nymphal instars (Fig. 2). Similar results were observed by Brisson et al. (2010), who reported that the expression of *en*, *hh*, *dpp*, *ubx*, *ap*, *wg*, *hth*, and *dll* showed no significant differences between wing morphs of third instar nymphs in *A. pisum,* but did not examine expressions of *vg*, *sal*, *omb*, and *srf*. Although the expression levels of *en*, *hh*, *sal*, *wg*, *exd*, and *Ubx* were found to be significantly different between macropterous (migratory) and brachypterous forms of *Nilaparvata lugens* [38], our study showed that there were no significant differences in the expressions of these genes between aphid morphs at the third nymphal instar. There is the possibility that brachypterous *N. lugens* adults still have short wings, while wingless aphids have no wings which degenerate by the second nymphal instar during development [38, 39]. This suggests that different developmental divergence times likely require different molecular mechanisms.

All aphids are born with wing buds, and they degenerate in the unwinged morphs during the second instar. In contrast, the wing buds continue to develop in the winged morphs [24]. Our results showed the expression of *vg* was highest in wingless adult aphid during development, while the expression of the *vg* gene was highest in third instar nymphs of the winged aphids. Third instar nymphs showed the greatest difference expressions of *vg* (21.38-fold) during development compared to fourth instar nymph and adult between the wing morphs, while *vg* expression levels showed no significant difference in first and second instar nymphs (Fig. 3). It is worth noting that we were unable to distinguish winged from wingless first or second instar nymphs by external morphology. The winged vs. wingless samples at these stages contain the opposite morph at 40% possibility (see methods). The different expression of *vg* between winged and wingless morphs at these two stages are likely underestimates of true differences. Altogether, it is possibility that wing discs degenerate in the wingless lines owing to the lack of *vg* expression.

 Gene expression can be regulated by both transcriptional and post-transcriptional mechanisms. Transcriptional regulation is often determined by *cis*-elements located within a gene’s promoter as well as by the epigenetic status of the gene and the adjacent DNA sequences [40]. The expression of *vg* during wing development may be regulated by *wg*, *dpp*, and *su(h)* interacting with *vg* enhancers in *Drosophila* [41, 42, 35]. However, no differences in expression levels for *wg*, *dpp*, and *su* (*h*) between the wing morphs in our study (Fig. 2). Therefore, we hypothesized that *vg* expression may be post-transcriptionally regulated by MicroRNAs (miRNAs). miRNAs are endogenous non-coding RNAs that post-transcriptionally regulate transcript levels and translational status of mRNA by degrading mRNA or terminating translation [43]. miRNA also may stable mRNA by mRNA-miRNA interactions [44]. miRNAs have been shown to regulate a variety of physiological and pathological processes throughout insect development including molting, metamorphosis, oogenesis, embryogenesis, behavior, and host-pathogen interactions [45]. However, few studies have investigated the potential role of miRNAs in wing polyphenism. Yang et al. (2014) found that miR-133 controls dopamine synthesis to control the production of solitarious versus gregarious forms in *Locusta migratoria*, however, direct evidence showing that miRNAs regulate wing development in aphids has yet to be reported. At present, there are few studies describing how miRNAs regulate expression of the *vg* gene. We used bioinformatics to predict that miR-147b could potentially regulate the expression of *vg*. In humans, miR-147b regulates some cellular effects including proliferation, migration, and apoptosis [46]. Importantly, miR-147b is involved in endothelial barrier function and is a potent inducer of intestinal epithelial cell differentiation [47, 48]. We found that *vg* expression was reduced and wing development was repressed after injecting the miR-147b mimic into winged lines at third instar nymph. This is consistent with the target experiments in which the co-transfection of miR-147b mimics with the corresponding target plasmids significantly decreased the relative luciferase activity. Our results provide direct evidence that miR-147b-meditated regulation of *vg* expression controls wing development in *R. padi*.

Although we determined here that *vg* plays an important role in wing development of *R. padi*, wing polyphenism is involved in both initial determination and subsequent differentiation [49]. Currently, some studies have indicated that neuroendocrine signaling pathways regulate wing morph determination [6, 7, 50]. For example, in *Nilaparvata lugens*, two insulin receptors regulate wing bud development by responding to an insulin-like peptide secreted by the brain, and produce long-winged or short-winged forms [51]. In *A. pisum*, Vellichirammal et al. [8] and Grantham et al. [50] proposed a wing morph determination hypothesis in which maternally supplied ecdysone affects embryonic insulin signaling, which ultimately plays a role in alternative morph development. These suggest the endocrine signaling (e.g. ecdysone and insulin) may regulate the miR-147b or *vg* expressions to control wing polyphenism in aphids. The point needs to be determined in the future.

## Conclusions

In summary, of the 20 genes involved in major wing development events, only *vg* shows significantly different expression levels between wingless and winged third nymphs. The *vg* plays an important role in wing development confirmed by *v*g RNAi. Also, *vg* transcription is regulated by miR-147b, which binds to its target sequence present in the *vg* mRNA. These results provide an evidence that miR-147b –modulated *vg* expression regulates the wing development in *R. padi*.

## Methods

### Insects and cell line culture

Colony of *Rhopalosiphum padi* was collected from a wheat field at the Agricultural Experiment Station of China Agricultural University (N40°03′, E116°28′) in May 2005 [52]. The stock parthenogenetic colony was derived from a single apterous female from the colony and maintained >10 generations at low density (~10 aphids per plate) to get rid of the telescoping effects of generations in which adult parthenogenetic aphids carry not only their daughters but also some of their granddaughters within them. Both wing morphs were induced by manipulating the adult density. Specifically, the stock parthenogenetic colony was divided into two groups. For the high-density condition to induce the winged morph, >30 adult wingless aphids were reared on wheat seedlings in each plastic peri dish (9 cm diameter, 20 cm tall), and the induction ratio of winged aphids in next generation under HD conditions was 43.0% ± 17.4% (*n* = 300 ± 38.4). Under the low-density (LD) condition, only one wingless adult was reared on wheat seedlings, and 100% (*n* = 63 ± 4.8) wingless aphids were induced. The aphids were reared in plastic petri dishes containing wheat seedlings in a climate controlled chamber under the following conditions: a temperature of 22 ± 1 °C, relative humidity of 50 ± 10%, and a photoperiod of 16 h:8 h (day:night). All of the wingless morphs used in our study were obtained from the LD condition, and the winged morphs were induced under HD conditions except for the effect of density on gene expression in which the wingless morphs from HD conditions are also used.

The mammalian HEK293T cell line was a gift from Institute of Microbiology, Chinese Academy of Sciences and maintained at 37 °C under a 5% CO_2_ atmosphere in DMEM high-glucose medium (Gibco, Grand Island, USA) containing 10% fetal bovine serum (Gibco).

### RNA extraction and cDNA synthesis

Because the third instar is the earliest stage when the wing morphs can be distinguished by examining outer morphology and the body wall is the part where the wing buds extend. To determine whether wing development genes were differently expressed between wing morphs, two types of aphid samples were prepared from third instar wingless and winged aphids for total RNA extraction: 1) whole bodies of 20 aphids, 2) various body parts (head, body wall and body cavity) of 50 aphids. Body parts were dissected from aphid under a binocular microscope. Specifically, we placed the aphid supine on a rubber tray, anchored it by carefully piercing the posterior edge of the abdomen, and used the dissecting knife cut its head as the head sample. Next, we peeled the venter of the abdomen off using the tip of another pin or knives and obtained the inside liquid tissues as the body cavity sample. The remaining part was washed in cold phosphate-buffered saline (PBS: 130 mM NaCl, 7 mM Na_2_HPO_4_•2H_2_0, 3 mM NaH_2_PO_4_•2H_2_O; pH 7.0), then removed excess water using paper as the body wall sample. Here, the body wall was considered as enriching in tissues containing cells to develop wing in winged aphid. To investigate the expression levels of *vg* between wing morphs at different developmental stages, body walls of 20 aphids from each instar and adult in each wing morph were collected for RNA extraction.

Total RNA was isolated using Trizol reagent (Invitrogen, USA) according to the manufacturer’s instructions. An additional DNaseI digestion was performed using RNase-Free DNaseI (Takara, Dalian, China). First-strand cDNA synthesis was carried out with a Reverse Transcription System (Takara) according to the manufacturer’s instructions.

Small RNAs were isolated from aphids using the miRNeasy Mini Kit (Qiagen, Germany) following the manufacturer’s protocol. First-strand cDNA was synthesized from 2 μg of total RNA using the miScript II RT kit (Qiagen) as directed by the manufacturer.

### Quantitative real-time PCR (qRT-PCR)

qRT-PCR was performed on an ABI 7500 Fast Real-Time PCR System (Applied Biosystems) using SYBR® Premix Ex Taq™ II (Tli RNaseH Plus) kit (Takara, Japan). The cycling program for qRT-PCR assays for miRNA or mRNA was as follows: initial incubation at 50 °C for 2 min and then at 95 °C for 2 min, followed by 40 cycles of 95 °C for 15 s and 60 °C for 30 s according to the manufacturer’s protocol. Analysis of the qRT-PCR data was carried out using the 2^−∆∆Ct^ method of relative quantification. As an endogenous control, the EF-1α and U6 snRNA transcripts were used to normalize the expression level of mRNA (or DNA) and miRNA, respectively [53, 54]. RT-qPCR plates were set up with three cDNA biological replicates and two technical replicates of each biological replicate. Samples for three biological replicates were collected over at least 2 days and two plastic petri dishes for wheat aphid culture. All primers in the study were designed based on information from a transcriptome library (PRJNA555831) of *R. padi* and were listed in Additional file 1: Table S1.

### Cloning and sequence analysis of *vg* cDNA

qRT-PCR results showed that *vg* expression levels were significantly higher in the winged aphids relative to wingless aphids, so we cloned and sequenced *vg* cDNA to examine its role in wing development. Specifically, total RNA from a mixed sample consisting of 60 aphids from various developmental stages and morphs was isolated as described above. For amplification of a partial *vg* cDNA sequence, PCR primers were designed based on information from the transcriptome library (PRJNA555831) of *R. padi*. The 5′- and 3′-ends of the cDNA molecules were amplified using the rapid amplification of cDNA ends method with the Gene-RACE Kit (Takara Biotechnology, Dalian, China) following the manufacturer’s instructions. BLAST searches for homologous sequences and the prediction of conserved regions were performed on the National Center for Biotechnology Information (NCBI) website (https://blast.ncbi.nlm.nih.gov/Blast.cgi).

### *vg* gDNA quantification

qRT-PCR results showed *vg* mRNA expressions were significantly higher in body walls of winged aphids relative to wingless aphids at the third nymphs. *A. pisum* genome shows a large number of gene duplications [55]. So, we determined if gene DNA copy number contributes to the difference by using qRT-PCR: the genomic DNA was isolated from body wall of 20 third instar of wing morphs using DNAzol (MRC) according to the manufacturer’s instructions. qRT-PCR were performed as described above, except that the primers were designed based on the *vg* exon sequences which were confirmed by aligning *vg* ORF nucleotide sequences with *A. pisum* genome in NCBI.

### Western blotting

Total proteins were extracted from 300 body walls of third instar nymphs by using 1× SDS-PAGE loading buffer (diluted by 1× PBS buffer, pH 7.5). A total of 30 μg of protein was loaded onto an SDS-polyacrylamide gel. After electrophoresis under 100 V for 2 h, protein was transferred to polyvinylidene difluoride membranes (Millipore, USA) under 100 mA for 20–30 min. Blots were then blocked in TBST (0.1% Tween 20 in TBS, pH 8.0) and 5% nonfat powdered dry milk (w/v) for 2 h. The blot was then probed using primary antibodies against *vg* protein at a dilution of 1:1000 in TBST with 5% nonfat powdered dry milk by incubating for 2 h. After the membrane was washed with TBST three times for 10 min each time, the membranes were incubated with horseradish peroxidase-conjugated secondary antibodies (Jackson ImmunoResearch, West Grove, PA, USA) at a dilution of 1:20000 in TBST for 30 min. After three additional washes with TBS, immunolabeled bands were detected by Immobilon Western Chemiluminescent HRP Substrate (Millipore Sigma, USA). Protein bands were scanned (Bio-Rad, Hercules, CA, USA). All was performed at room temperature.

The antibodies used in this study were purchased from Abiotech (Jinan, China). The *vg* antibody preparation was conducted as follows: the open reading frame of the *vg* gene was inserted into the pET-16b expression vector. The resulting recombinant vector was transformed into *Escherichia coli* BL21 cells, and expression was induced with 1 mM isopropyl β-D-1-thiogalactopyranoside (IPTG). The produced fusion protein was identified by 15% sodium dodecyl sulphate polyacrylamide gel electrophoresis (SDS-PAGE) and further purified using His-Bind resin (Ni^2+^-resin; Novagen, Germany) according to the manufacturer’s protocol. The purified protein (100 μg) in complete Freund’s adjuvant was injected subcutaneously to immunize New Zealand white rabbits, followed by two booster injections (200 μg) in incomplete Freund’s adjuvant. One week after the last injection serum was collected, separated and stored at− 20 °C for immunoassays.

### RNA interference (RNAi)

The specific primers containing a T7 polymerase promoter sequence were designed on E-RNAi (http://www.dkfz.de/signaling/e-rnai3/). The specific primers were used to amplify the fragments of *vg* using reverse transcription PCR (RT-PCR). A 486 bp fragment of *vg* was used as the template for dsRNA synthesis using the TranscriptAid T7 High Yield Transcription Kit (Thermo Scientific, Wilmington, DE, USA) synthesis following the manufacturer’s instructions. The dsRNA of enhanced green fluorescent protein (EGFP) was used as a control. All of the synthesized dsRNAs were dissolved in nuclease-free water and then quantified using a NanoDrop 2000 (Thermo Scientific, Wilmington, DE, USA), and stored at − 20 °C until use.

dsRNA-*vg* of approximately 13.8 nL (1000 ng/μL) were injected into thorax segments of third instar winged aphids using a micro-injector (Nanoliter 2000 Injector, WPI Inc. Sarasota, FL, USA). Controls were injected with dsEGFP. More than 100 injected aphids were placed on wheat seedlings to recover and were then reared under laboratory conditions. A total of 20 injected aphids were randomly collected at 24 h post-injection for the subsequent detection of *vg* expression using qRT-PCR. The remaining insects were maintained for observation of their phenotypes and growth status. Photos were taken with a Leica M165C microscope (Leica Microsystems, Wetzlar, Germany) at 48 h after injection. All experiments were independently repeated at least three times.

### miRNA target studies of *vg*

To determine whether *R. padi* miRNA could target *vg*, two commonly miRNA target prediction programs (miRanda (http://www.microrna.org/microrna/getDownloads.do) and RNAhybrid (http://bibiserv.techfak.uni-bielefeld.de/rnahybrid/welcome.html)) and one miRNA library of *R. padi* (PRJNA555833) were used. The predicted miRNAs were selected to investigate their expression levels between third instar wingless and winged aphids using RT-qPCR. A total of 20 aphids were used as a biological replicate, and three replicates were performed.

### Dual luciferase reporter (DLR) assay

The agomir (mimic) of miR-147b was designed and synthesized by GenePharm Co. Ltd. (Shanghai, China). The miRNA agomir is a dsRNA form from the miRNA and its complimentary sequence with a chemical modification. The negative control was designed based on a *Caenorhabditis elegans* miRNA with no similarity to insect miRNAs. Two 226-bp fragments containing the miR-147b predicted target sites and the mutated miR-147b target DNA sequence were amplified by PCR and inserted downstream of the luciferase gene in the pmirGLO vector (Promega, USA) between the PmeI and XhoI restriction sites to give the pmirGLO-miR-147b and pmirGLO-miR-147b-mut target constructs. The dual luciferase reporter (DLR) assay was performed as previously described [54]. HEK293T cells were cultured in a 24-well plate and transfected with the target plasmids and either the miRNA agomir or NC using the Calcium Phosphate Cell Transfection Kit (Beyotime, Nanjing, China) according to the manufacturer’s instructions. Each well contained 0.2 μg plasmid DNA with 100 nM final concentration of the miRNA agomir. Luciferase assays were performed using the Dual-Glo® Luciferase Assay System (Promega) 24 h post-transfection. Normalized firefly luciferase activity (firefly luciferase activity/*Renilla* luciferase activity) was compared to that of the control pmirGLO Vector. The mean of the relative luciferase expression ratio (firefly luciferase/*Renilla* luciferase) of the control was set to 1. For each transfection, the luciferase activity was averaged from five replicates.

### Modulation of miRNA and the subsequent impacts on wing development

Each aphid was injected with 13.8 nL of a 40 μM agomir solution, and the control was injected with agomir-NC in third instar winged aphids. At 24 h post-injection, the 20 nymphs in each sample were collected for later detection of gene expression. The relative expression levels of *vg* and miR-147b were determined using qRT-PCR. The remaining insects were maintained for observation of their phenotypes after injection 48 h. All experiments were performed in triplicate.

### Statistical analysis

Independent samples analysis of Student’s t-test was used to compare the relative expression of each wing development gene (or miR-147b) between the wingless and winged morphs, between the dsRNA treatment groups and the control, and between miR-147b agomir treatment groups and the control. One-way analysis of variation (ANOVA) followed by Tukey’s multiple comparisons was used to compare the relative expression of *vg* transcript in different tissues or development stages (tested data were normally distributed). All the statistical analysis was conducted using the SPSS software v. 20. A *P*-value < 0.05 was considered to be statistically significant.

## Supplementary information

**Additional file 1: Figure S1.** Multiple alignment of the Vg_Tdu domains from *vg* proteins from 18 insect species. * indicates conserved amino acids in the different insect sequences. Protein sequences were from *Myzus persicae* (XP_022168953); *Acyrthosiphum pisum* (XP_003242605); *Diuraphis noxia* (XP_015367189); *Tribolium castaneum* (XP_008199328); *Dendroctonus ponderosae* (XP_019757352); *Drosophila melanogaster* (AAB20671); Lucilia cuprina (XP_023292552); *Musca domestica* (XP_005187398); *Nilaparvata lugens* (XP_022194027); *Blattella germanica* (CUT08830); *Bemisia tabaci* (XP_018900015); *Solenopsis invicta* (XP_011161686); *Acromyrmex echinatior* (XP_011053596); *Apis mellifera* (XP_016771047); *Megachile rotundata* (XP_012136065); *Neodiprion lecontei* (XP_015514063); *Bombyx mori* (XP_012545611). **Figure S2.** Western blot analysis of VG protein in body walls of third instar wingless (3rdWL) and winged (3rdWD) morphs. β-actin was used as the internal control. **Figure S3.** The DNA expression levels of *vg* in the body walls of third wingless (3rdWL) and third winged (3rdWD) morphs of *R. padi***.** Data presented as the mean ± SD for three independent replicates. **Figure S4.** Expression levels of *vg* and miR-147b in the body walls of third instar wingless nymph (3rdWL) from low-density (LD) and high density conditions. 3rdWL-LD were obtained from a single wingless adult female that was reared on wheat seedlings, and 100% wingless aphids were produced. 3rdWL-HD were produced under conditions of crowding, where > 30 adult wingless aphids were reared on wheat seedlings in plastic petri dishes, and the percentage of winged aphids was 43.0 ± 17.4%. **Table S1.** Primers and nucleotides used in experiments.

## Data Availability

The cDNA sequences from the study has been deposited in GenBank under the accession number MH168385. The raw data of transcriptome library and miRNA library used in this study have been deposited in the NCBI Short Read Archive under PRJNA555831 and PRJNA555833.

## Abbreviations

A-P: Anterior-posterior
D-V: Dorsal-ventral
miRNAs: microRNA
ORF: Open reading frame
qRT-PCR: Quantitative real-time reverse transcription polymerase chain reaction
RNAi: RNA interference
UTR: Untranslated region
