# Supplementary material for: miR-147b-modulated expression of vestigial regulates wing development in the bird cherry-oat aphid Rhopalosiphum padi
Source: BMC Genomics. 2020 Jan 22;21:71. doi: 10.1186/s12864-020-6466-7 (PMC6977242; doi:10.1186/s12864-020-6466-7)
Supplement: Supplementary file 1 — Additional file 1: Figure S1. Multiple alignment of the Vg_Tdu domains from vg proteins from 18 insect species. * indicates conserved amino acids in the different insect sequences. Protein sequences were from Myzus persicae (XP_022168953); Acyrthosiphum pisum (XP_003242605); Diuraphis noxia (XP_015367189); Tribolium castaneum (XP_008199328); Dendroctonus ponderosae (XP_019757352); Drosophila melanogaster (AAB20671); Lucilia cuprina (XP_023292552); Musca domestica (XP_005187398); Nilaparvata lugens (XP_022194027); Blattella germanica (CUT08830); Bemisia tabaci (XP_018900015); Solenopsis invicta (XP_011161686); Acromyrmex echinatior (XP_011053596); Apis mellifera (XP_016771047); Megachile rotundata (XP_012136065); Neodiprion lecontei (XP_015514063); Bombyx mori (XP_012545611). Figure S2. Western blot analysis of VG protein in body walls of third instar wingless (3rdWL) and winged (3rdWD) morphs. β-actin was used as the internal control. Figure S3. The DNA expression levels of vg in the body walls of third wingless (3rdWL) and third winged (3rdWD) morphs of R. padi. Data presented as the mean ± SD for three independent replicates. Figure S4. Expression levels of vg and miR-147b in the body walls of third instar wingless nymph (3rdWL) from low-density (LD) and high density conditions. 3rdWL-LD were obtained from a single wingless adult female that was reared on wheat seedlings, and 100% wingless aphids were produced. 3rdWL-HD were produced under conditions of crowding, where > 30 adult wingless aphids were reared on wheat seedlings in plastic petri dishes, and the percentage of winged aphids was 43.0 ± 17.4%. Table S1. Primers and nucleotides used in experiments. [file 12864_2020_6466_MOESM1_ESM.docx]

**miR-147b-modulated expression of *vestigial* regulates wing development in the bird cherry-oat aphid *Rhopalosiphum padi***

Yinjun Fan, Xiuxia Li, Abd Allah A. H. Mohammed, Ying Liu, Xiwu Gao

Department of Entomology, China Agricultural University, Beijing, China.

Correspondence: Xiwu Gao, Department of Entomology, China Agricultural University, No. 2 Yuanmingyuan West Road, Haidian District, Beijing 100193, P.R. China.


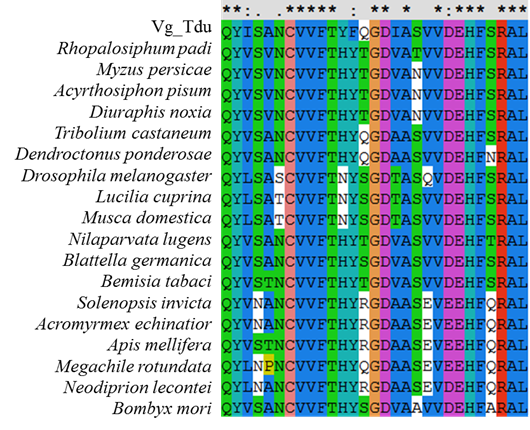


**Fig. S1. Multiple alignment of the Vg_Tdu domains from *vg* proteins from 18 insect species**. * indicates conserved amino acids in the different insect sequences. Protein sequences were from *Myzus persicae* (XP_022168953); *Acyrthosiphum pisum* (XP_003242605); *Diuraphis noxia* (XP_015367189); *Tribolium castaneum* (XP_008199328); *Dendroctonus ponderosae* (XP_019757352); *Drosophila melanogaster* (AAB20671); Lucilia cuprina (XP_023292552); *Musca domestica* (XP_005187398); *Nilaparvata lugens* (XP_022194027); *Blattella germanica* (CUT08830); *Bemisia tabaci* (XP_018900015); *Solenopsis invicta* (XP_011161686); *Acromyrmex echinatior* (XP_011053596); *Apis mellifera* (XP_016771047); *Megachile rotundata* (XP_012136065); *Neodiprion lecontei* (XP_015514063); *Bombyx mori* (XP_012545611).

**
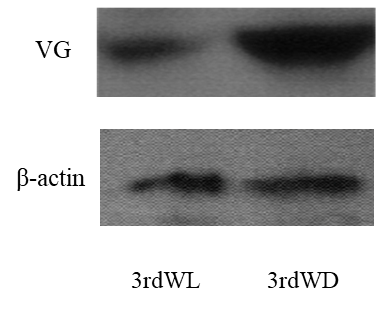
**

**Fig. S2. Western blot analysis of VG protein in body walls of third instar wingless (3rdWL) and winged (3rdWD) morphs.** β-actin was used as the internal control.


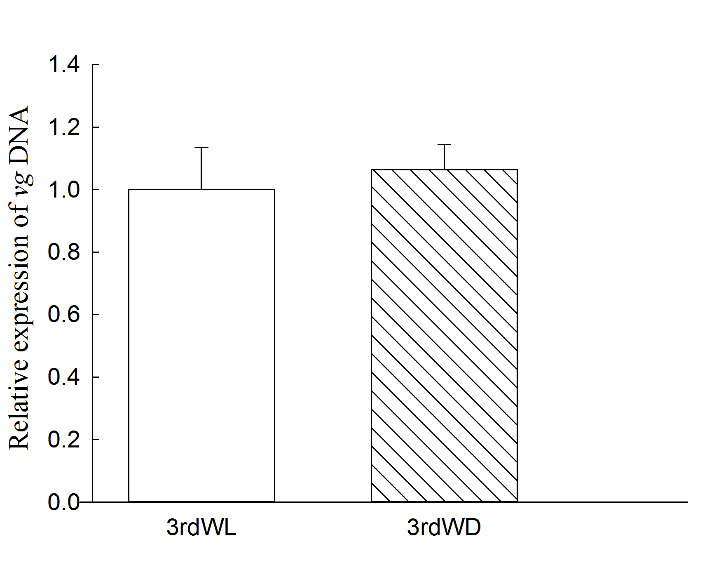


**Fig. S3. The DNA expression levels of *vg* in the body walls of third wingless (3rdWL) and third winged (3rdWD) morphs of *R. padi*.**

**
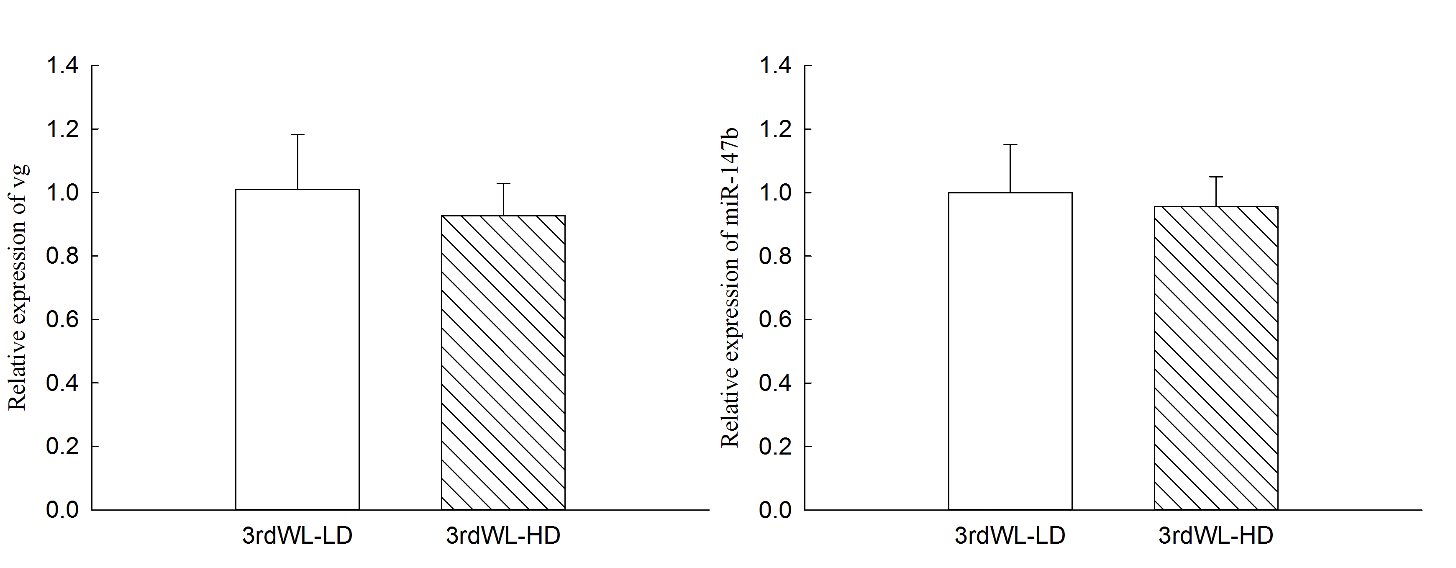
**

**Fig. S4. Expression levels of *vg* and miR-147b in the body walls of third instar wingless nymph (3rdWL) from low-density (LD) and high density (HD) conditions**. 3rdWL-LD were obtained from a single wingless adult female that was reared on wheat seedlings, and 100% wingless aphids were produced. 3rdWL-HD were produced under conditions of crowding, where >30 adult wingless aphids were reared on wheat seedlings in plastic petri dishes, and the percentage of winged aphids was 43.0±17.4%.

**Table S1. Primers and nucleotides used in experiments.**

| Primer name | Sequence (5’-3’) | Application |
| --- | --- | --- |
| EF1α-F | TAGACGCTATCCTACCCCCCA | Real-Time PCR |
| EF1α-R | GTGAAATCAGCAGCACCCTTG | Real-Time PCR |
| wg-F | ATTGTGGACAGAGGTTTCAGGGAG | Real-Time PCR |
| wg-R | GCCCCACTCCCAGTCCTTGA | Real-Time PCR |
| hh-F | TAATCGGCATTCACAAGCAACC | Real-Time PCR |
| hh-R | ACCAGCGGCTTCATCTTTCG | Real-Time PCR |
| brk-F | GAACCCGTCGGCAAGGTGGAAG | Real-Time PCR |
| brk-R | CGTGGCTCGCTGATTTCCCTGG | Real-Time PCR |
| ap1-F | TCGTCGAACTCAGACTTGAAGGTGC | Real-Time PCR |
| ap1-R | ATGGGCGTGTTTGAAGACCTGG | Real-Time PCR |
| ap2-F | CAACGTGCTCAGCATACCACAATC | Real-Time PCR |
| ap2-R | GACAGTCCAGTTTTTTGAGCCAGC | Real-Time PCR |
| dpp-F | GCCGTGGCAGTAGTAAGCGTCGTAG | Real-Time PCR |
| dpp-R | TGGCAAGAATAGCCGTCCCGTC | Real-Time PCR |
| Ubx-F | CGCTCGTCATACAGAATCGTCCCA | Real-Time PCR |
| Ubx-R | AATGTGTGGTGTGAGTGTGTGCGG | Real-Time PCR |
| vg-F | AGAGACGATGCTCGCACTGAAA | Real-Time PCR |
| vg-R | TTCTGGAAGACATTGGGGAAGTAT | Real-Time PCR |
| dll-F | CTAAGGATGCGGCAAGTTCAG | Real-Time PCR |
| dll-R | GTGGTATCGCAGAAGATGGCA | Real-Time PCR |
| sd-F | CGTTGGTGTTCCATAAATGCTGAGA | Real-Time PCR |
| sd-R | AACCTCCATCAACTGCTGTATCGTCT | Real-Time PCR |
| N-F | CAACCCACCAGGGAGTCTCTCATTA | Real-Time PCR |
| N-R | AAACGACATAGACCCGCTCCGATA | Real-Time PCR |
| dl-F | AACGCAACGCATCGCAGAGAA | Real-Time PCR |
| dl-R | AGTGCGTTTTTATCGTCTACGGGTGT | Real-Time PCR |
| ser-F | TGTTAGTGCCCAAGCAGTTCCA | Real-Time PCR |
| ser-R | CAACCCCTTCGACATACACATTCT | Real-Time PCR |
| su(h)-F | TGCCAGTTCAACAGAGTGGGGAG | Real-Time PCR |
| su(h)-R | TAGTCGGGGTAAAGCCATTCCAGA | Real-Time PCR |
| en2-F | CCATCACAGACACTACCACCCCCA | Real-Time PCR |
| en2-R | GCGGCGTTGTGACTGAGTTGGTT | Real-Time PCR |
| sal-F | GTCCCGCCGAAAGCAATCCA | Real-Time PCR |
| sal-R | GACAACGACAGCCGCACCCG | Real-Time PCR |
| srf-F | GTGCTACGAACCCACCGACC | Real-Time PCR |
| srf-R | GCCCTTGGTTTTCTTGCCGT | Real-Time PCR |
| hth-F | GCGGATTCAACATCGCACAC | Real-Time PCR |
| hth-R | CTCGCCTTTGGCACCATTTA | Real-Time PCR |
| omb-F | CAAACCGCTGCCGCTATGAC | Real-Time PCR |
| omb-R | GCCTGCTGCTGGACTGTAGACG | Real-Time PCR |
| exd-F | ATTGCTGAAGGAGTGGCTGGTC | Real-Time PCR |
| exd-R | TGCTCTGTAGTCGGAATGCTCG | Real-Time PCR |
| vgp1-F | CATCACTTCACGCCGTTCACC | Partial sequence |
| vgp1-R | TGAGTAAGACGTGCCAGTGTCCAAG | Partial sequence |
| vg5’-RACE outer | TGGGAAGTTTCTGGAAGACATTGG | 5’-RACE |
| vg5’-RACE inner | TCTCTGGATGTGTCCTCTTCCTTGT | 5’-RACE |
| vg3’-RACE outer | ATATGAGTGTCTCACCACTTCTGCG | 3’RACE |
| vg3’-RACE inner | GCCCAGTACGGCAGCCTATTA | 3’RACE |
| vgfull-F | GTCCCCGCGCTCACGTAC | Full-length |
| vgfull-R | GGAGACCGCAGAAGTGGTGAGA | Full-length |
| eGFP-F | taatacgactcactatagggagaCAGTGCTTCAGCCGCTAC | dsRNA synthesis |
| eGFP-R | taatacgactcactatagggagaGTTCACCTTGATGCCGTTC | dsRNA synthesis |
| dsvg-F | taatacgactcactatagggCGTGTCAGTAAATTGCGTGG | dsRNA synthesis |
| dsvg-R | taatacgactcactatagggCACGGTTCCATACCAGCTTT | dsRNA synthesis |
| qdsvg-F | GTTGAGAGCGTTGGAAACACACC | *vg*RNAi analysis |
| qdsvg-R | ACTGCTGTGATGTGAGTCCGTATG | vgRNAi analysis |
| vg-ORF-F | ATGAGCTGCACAGAGGTTATGTATC | VG analysis |
| vg-ORF-R | TTAAAACCAATATAGATCTTTGGACGTC | VG analysis |
| vg-DNA-F | CATACGGACTCACATCACAGCAG | Real-Time PCR |
| vg-DNA-R | GTACTACCGAAACGCTATTGGCT | Real-Time PCR |
| miR-147b-F | GTGTGCGGAAATGCTTCTGCT | Real-Time PCR |
| U6-F | CGCAAGGATGACACGCAA | Real-Time PCR |
| miR-R | GAATCGAGCACCAGTTACGC | Real-Time PCR |
| miR-147b-F | GTTTAAACTCATACGGACTCACATCACAGCAG | miRNA function |
| miR-147b-R | CTCGAGGTGTGCGGCAACACGCCT | miRNA function |
| miR-147b-mut-R | CTCGAGACTGTATACAACACGCCTGTCCCCGC | miRNA function |
